# Re-evaluating the genotypes of patients with adenomatous polyposis of unknown etiology: a nationwide study

**DOI:** 10.1038/s41431-024-01585-z

**Published:** 2024-03-12

**Authors:** John Gásdal Karstensen, Thomas v. Overeem Hansen, Johan Burisch, Malene Djursby, Helle Højen, Majbritt Busk Madsen, Niels Jespersen, Anne Marie Jelsig

**Affiliations:** 1 Danish Polyposis Register, Gastro Unit, Copenhagen University Hospital - Amager and Hvidovre, Hvidovre, Denmark; 2 Department of Clinical Medicine, University of Copenhagen, Copenhagen, Denmark; 3 Department of Clinical Genetics, Copenhagen University Hospital - Rigshospitalet, Copenhagen, Denmark; 4 Gastrounit, Medical Division, Copenhagen University Hospital - Amager and Hvidovre, Hvidovre, Denmark; 5 Center for Genomic Medicine, Copenhagen University Hospital - Rigshospitalet, Copenhagen, Denmark

**Keywords:** Genetics research, Epidemiology

## Abstract

In the Danish Polyposis Register, patients with over 100 cumulative colorectal adenomas of unknown genetic etiology, named in this study colorectal polyposis (CP), is registered and treated as familial adenomatous polyposis (FAP). In this study, we performed genetic analyses, including whole genome sequencing (WGS), of all Danish patients registered with CP and estimated the detection rate of pathogenic variants (PV). We identified 231 families in the Polyposis Register, 31 of which had CP. A polyposis-associated gene panel was performed and, if negative, patients were offered WGS and screening for mosaicism in blood and/or adenomas. Next-generation sequencing (NGS) was carried out for 27 of the families (four declined). PVs were detected in 11 families, and WGS revealed three additional structural variants in *APC*. Mosaicism of a PV in *APC* was detected in two families. As the variant detection rate of eligible families was 60%, 93% of families in the register now have a known genetic etiology.

## Introduction

Familial adenomatous polyposis (FAP) is the most common hereditary polyposis syndrome and has been known for over a century [1]. In its classical form, it is characterized by the development of multiple colorectal adenomas during adolescence that, if left untreated, develop into colorectal cancer [2]. Patients also have a high risk of upper-GI involvement such as duodenal polyps and cancer (Supplementary Material 1) [3].

In 1991, heterozygous pathogenic variants (PV) in adenomatous polyposis coli (*APC*) were identified as the cause of FAP, explaining the autosomal dominant inheritance pattern observed in families [4, 5]. *MUTYH* was identified as the first gene to cause an autosomal recessive inherited form of polyposis, named *MUTYH*-associated polyposis (MAP). This syndrome helped to explain other patients with colorectal adenomatous polyposis [6]. Recent advances in genetic sequencing technology, such as next-generation sequencing (NGS), have helped to identify several novel causes for hereditary adenomatous polyposis (Supplementary Material 2) [7]. It is important to differentiate patients with FAP from patients with other syndromes, as surveillance is tailored to specific genetic syndromes [8]. Furthermore, a genetic diagnosis is important for evaluating the risk among relatives. NGS now also includes the possibility of whole genome sequencing (WGS) to detect deep intronic variants and large rearrangements. NGS also makes it easier to screen for mosaicism in tissues other than blood [9].

The Danish Polyposis Registry was founded in 1971 and all patients diagnosed with FAP (of known genetic etiology) or colorectal polyposis (CP) of unknown genetic etiology are systematically registered. CP is defined as over 100 cumulative colorectal adenomas. In recent years, genetic diagnostics has increasingly been integrated into the care of these patients and has offered them the possibility of a precise diagnosis and reevaluation of the optimal disease surveillance. The purpose of the study was to systematically offer genetic analyses to registered families with CP and to evaluate the use of different sequencing methods and estimate the variant detection rate.

## Materials and methods

In April 2018, we systematically reviewed families registered in the Polyposis Registry and identified those with CP defined as a patient having more than 100 cumulative colorectal adenomas. No family members in any of these families had been genetically tested, or if they had the results were negative. The phenotypes of the families were also carefully evaluated. The families were included if:

1. the proband was alive and had CP.
2. the proband was deceased but had a diagnosis of CP and a living, first-degree relative.

Genetic testing was offered to a proband or affected family member. Primarily, genetic testing was performed on DNA extracted from peripheral blood using a NGS gene panel in one affected family-member. The panel was custom-made, and the combination of genes was continuously adjusted according to new discoveries, but the panel contained at least the following genes: *APC, BMPR1A, EPCAM, MLH1, MLH3, MSH2, MSH6, MUTYH, PMS2, POLD1, POLE, PTEN, SMAD4, STK11*. The latest version of the gene panel was a custom Twist Bioscience capture, sequenced on an Illumina NextSeq 550. Reads were mapped to the human reference genome GRCh38, and single nucleotide variants (SNV) (with variant allele frequency >20%) and copy number variations (CNV) were called using GATK HaplotypeCaller and GermlineCNVCaller, respectively [10], in the coding regions and +/−50 bp of the surrounding intronic regions. Variant annotation and filtration were performed in VarSeq 2.3.0 (Golden Helix). When no blood samples were available because all affected relatives were deceased, the analyses were performed on DNA from formalin-fixed, paraffin-embedded, non-neoplastic tissue from a living, affected family member.

If genetic testing was negative, selected patients were offered further genetic testing, including screening for mosaicism of *APC* variants in blood and/or tissue from an adenoma. If one or more PV in *APC* was detected in the adenoma, a second or third adenoma was tested for the PV(s). These SNV were called using GATK Mutect2, with no lower variant allele frequency threshold [10]. Variant annotation and filtration were, again, performed in VarSeq 2.3.0 (Golden Helix). If a PV was not detected, WGS (Illumina Technology) was performed using a NovaSeq 6000 system, as recently described [11]. The data were analyzed for SNV, genomic structural variants, and CNV using CNVkit, CNVnator, Manta, and Lumpy using VarSeq 2.2.3 (Golden Helix). If a PV was identified in one family member, we assumed that affected relatives had the same PV. In families where more than one family-member were affected and the NGS panel were negative, the index patient and an additional family-member was offered genetic testing with WGS to compare genetic data. Variants were classified according to general American College of Medical Genetics (ACMG) guidelines (for *MUTYH*, *NTHL1*, and *POLE*) or *APC* gene-specific ACMG guidelines [12, 13].

## Results

By April 2018, the Danish Polyposis Register comprised 231 families, 110 (47.6%) had FAP and 121 (52.4%) had CP. At that point, 31 families comprising 45 patients met the inclusion criteria of this study and were eligible for genetic testing or re-testing.

### Clinical characteristics

In all families, at least one patient had CP in the colon and in 22 families at least one family members had duodenal adenomas. In 21 families only one family member was affected.

### Genetic results

**Fig. 1:**
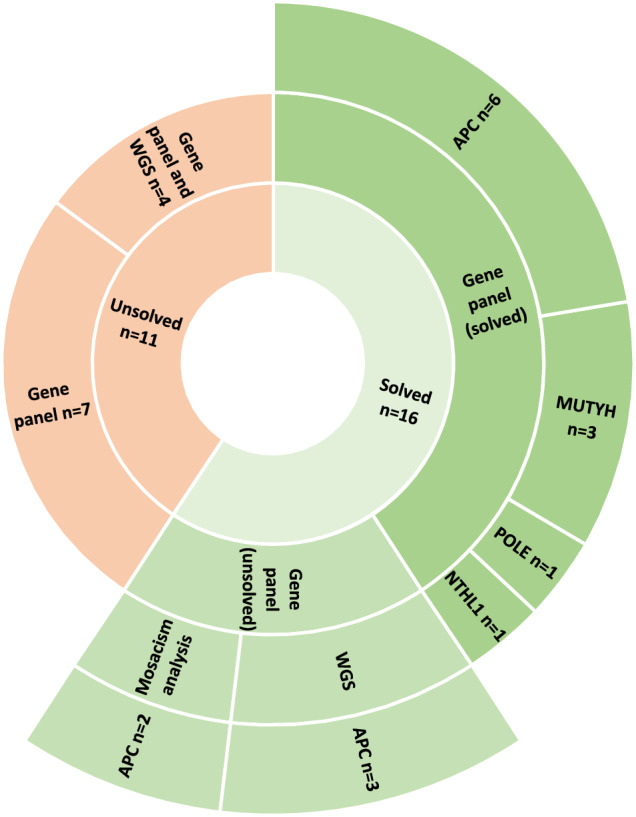
Results of genetic testing in patients with colorectal polyposis.

In four families with CP all family members declined genetic analysis, but a NGS panel was performed for members of the remaining 27 families. A PV was detected in 11 families, including six PV in *APC*, three homozygous PV in *MUTYH*, a homozygous PV in *NTHL1*, and a PV in *POLE*. When screening for mosaicism in blood and/or several adenomas, two additional pathogenic PV in *APC* were detected and were deemed to be likely representative of low-grade mosaicism. Both were from families with only one affected member. WGS was performed in seven families and showed three additional structural variants involving *APC* and were concluded to be pathogenic. The remaining families declined WGS. The results are shown in Fig. 1 and the variants detected are listed in Table 1. In total, we detected a PV in 16 out of 27 families (60%). In 11 families with CP, we were unable to identify a PV despite meticulous genetic analyses, which included testing for mosaicism and WGS in four of the families. In all but one family, only one family-members were affected (90%).

**Table 1:** Genetic results.

Gene	DNA	Protein	ACMG criteria used	Variant class	Remarks
*APC* NM_000038.6 and NM_001127511.2					
	c.(?_-220)_(165 + 1_166-1)del	p.(?)	PS4; PP1; PM2_Supporting	LP	Deletion of promoter 1B (NM_001354897.2)
	c.(220 + 1_221-1)_(8532_?)del		PVS1; PM2_Supporting	LP	Deletion of exon 4–16 mosaicism in blood
	c.(422 + 1_423-1)_(834 + 1_835-1)dup		PVS1; PM2_Supporting	LP	Duplication involving exon 5-8
	c.694 C > T	p.(Arg232Ter)	PVS1; PM2_supporting; PS4	P	
	c.3927_3931del	p.(Glu1309Aspfs*4)	PVS1; PS4_VeryStrong; PM6_VeryStrong (reviewed by expert panel); ClinVar Accession: SCV003836601.1	P	
	c.4108 A > T	p.(Lys1370Ter)	PVS1; PM2_supporting	LP	
	c.4348 C > T	p.(Arg1450Ter)	PVS1; PM2_Supporting	LP	Variant detected in three polyps (mosaicism), not in blood
	c.5826_5829del	p.(Asp1942Glufs*27)	PVS1; PM2_Supporting; PP1; PS4	P	
	Insertion of retrotransposon in intron 12*	p.(?)		LP	
	Inversion involving exon 1B*	p.(?)		LP	
	Insertion of retrotransposon in exon 16*	p.(?)		LP	
*MUTYH* NM_001128425.2					
	c.734 G > A,	p.(Arg245His)	PS3; PS4; PP3	P	Homozygous
	c.1187 G > A	p.(Gly396Asp)	PS3; PS4; PP3 PM1	P	Homozygous
	c.1437_1439del	p.(Glu480del)	PS3; PS4; PM1	P	Homozygous
*NTHL1 NM_002528.6*					
	c.268 C > T	p.(Gln90Ter)	PVS1; PS4	P	Homozygous
*POLE NM_006231.4*					
	c.1270 C > G	p.(Leu424Val)	PS4; PM1; PP1; PP3	P	Heterozygous

*P* Pathogenic, *LP* Likely pathogenic;

*Detected with whole genome sequencing.

The register is dynamic, meaning that newly diagnosed families continuously are included, but also that patients with CP diagnosed with another genetic etiology than *APC* are excluded. By August 2023, the number of registered families with either FAP or CP being eligible for genetic testing was 150 (comprising 413 patients), and a PV in *APC* was known in 139 of these families (93%).

## Discussion

In this study, we used the nationwide Danish Polyposis Registry to identify families with CP. Using NGS, including screening for mosaicism and WGS, it was possible to detect a PV in 60% of families. In total, 93% of families in the Danish Polyposis Register now have a known PV.

The results highlight the increased diagnostic yield made possible by supplementing a NGS gene panel with subsequent WGS and/or searching for mosaicism. While not all patients were analyzed with WGS, we detected a PV in approximately 40% of those who did undergo WGS; however, this does not preclude using a NGS panel as the first choice. WGS allows us to detect large rearrangements, as shown in this study and others [14, 15]. Mosaicism is not easily detected with WGS due to a generally lower coverage per nucleotide, which tends to favor a gene panel with higher coverage. However, it has been demonstrated that low-level mosaicism can be restricted to colonic tissue, including adenomas or carcinomas, and so genetic analysis could be targeted at these tissues [16, 17]. However, the interpretation of PV detected in *APC* in adenomas or carcinomas can prove challenging as somatic variants in *APC* are common in these tumors and because many *APC* variants have been reported, both somatically and germline. If a PV in *APC* has been detected in the first adenoma, it should be confirmed in additional samples, i.e., at least one, or preferably two, additional adenomas or, if appropriate, in leukocyte DNA. If the same PV is detected in several adenomas, it is unlikely to be a somatic variant restricted to those adenomas [9].

Our study also demonstrates that CP can have genetic causes other than PV in *APC* (Fig. 1). Thus, we detected PV in *MUTYH, NTHL1*, and *POLE* (Table 1). This shows that genetic testing is essential for offering a precise diagnosis and personalized surveillance, including additional surveillance at extraintestinal sites (Supplementary Material 2) [8, 18]. However, the study also demonstrates that genetic testing, even in genes that have been known for decades, has not been offered to all of the families that it should have been. In addition, it is worth noting that pathogenic variants in genes associated with hamartomatous polyposis syndromes, e.g., *BMPR1A*, *PTEN* and *SMAD4*, can also be detected in patients with adenomatous polyposis and, to a lesser extent, vice versa [19]. This emphasizes that a clinical diagnosis of a specific polyposis syndrome is virtually impossible, unless the phenotype is indisputable, such as in Peutz-Jeghers syndrome, where patients exhibit characteristic mucocutaneous pigmentations.

We found that the variant detection frequency was over 90% in families in the National Register; however, there is still a small proportion of patients with CP whose PV was not detected despite our considerable efforts. Most of the genetically unsolved patients were isolated cases (90%) with no other affected relatives. This could indicate that new polyposis syndromes have yet to be defined, perhaps with autosomal recessive inheritance. Fortunately, new candidate genes for polyposis are continually being proposed [20]. *APC* mosaicism that cannot be detected using the aforementioned analyses is another possible explanation for unsolved cases.

The strength of this study is its nationwide approach, made possible by the Danish Polyposis Register that provides a unique opportunity for research and systematic follow-up of patients and their families. A limitation of the study is that some patients declined genetic testing and WGS.

We conclude that genetic analyses are crucial for a precise diagnosis and tailored disease surveillance, as well as the accurate assessment of at-risk family members. Using a NGS panel, WGS, and screening for mosaicism, the PV detection frequency in families with CP is now over 90%.

### Supplementary information

Supplementary Material 1

Supplementary Material 2

## Data Availability

Anonymized and summarized data collected for the study will be made available to other researchers upon publication and following reasonable requests made to the corresponding author.

## Code availability

This study uses data from the Danish Polyposis Register. The data collected will be made available to other researchers upon publication and following reasonable requests to the corresponding author.
