# Supplementary material for: Re-evaluating the genotypes of patients with adenomatous polyposis of unknown etiology: a nationwide study
Source: Eur J Hum Genet. 2024 Mar 12;32(5):588–92. doi: 10.1038/s41431-024-01585-z (PMC11061120; doi:10.1038/s41431-024-01585-z)
Supplement: Supplementary file 1 — Supplementary Material 1 [file 41431_2024_1585_MOESM1_ESM.pdf]

Supplementary Material 1

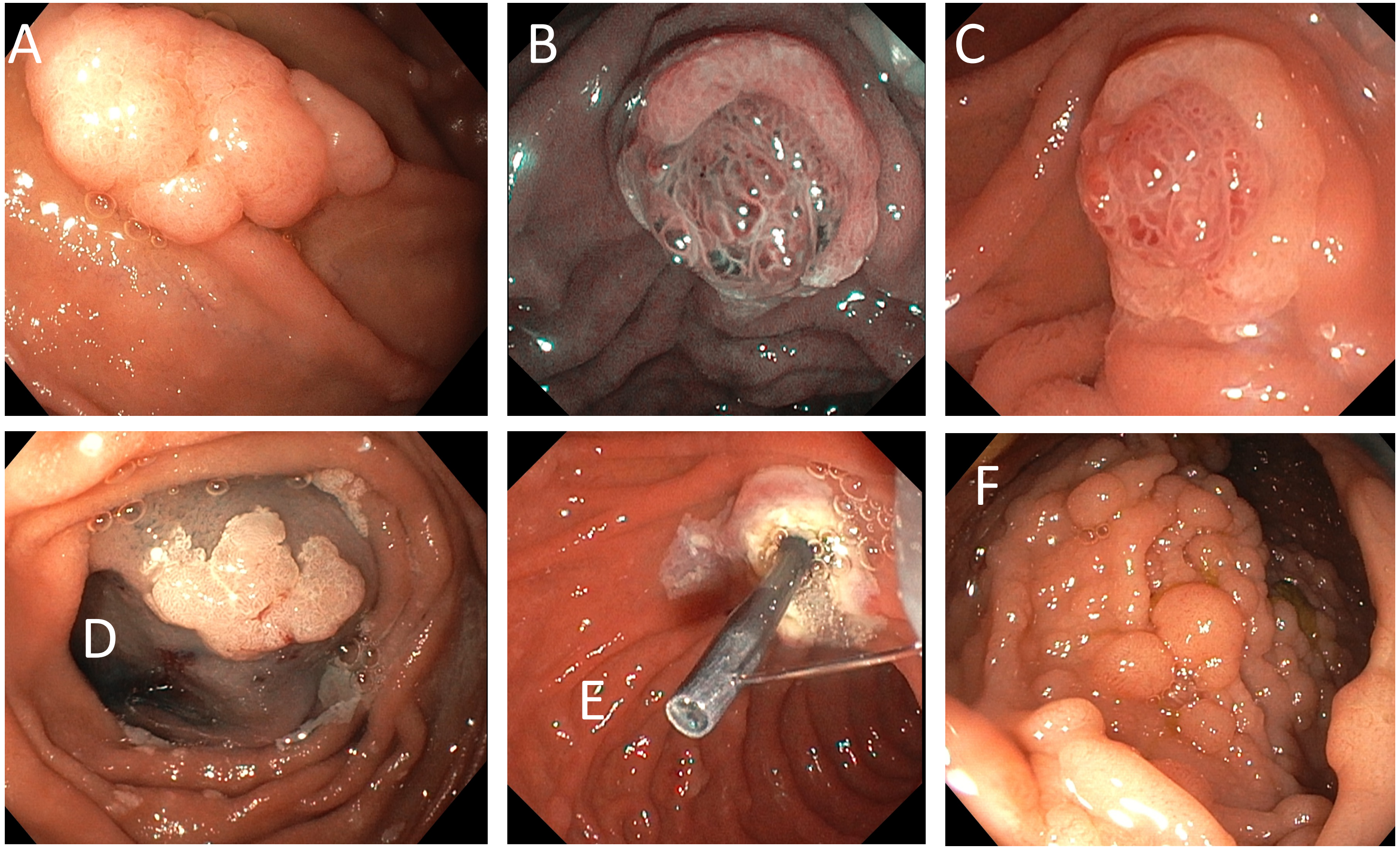

Endoscopic pictures demonstrating polyposis in a patient with FAP. A-E: Upper Gi-tract, F: Lower GI-tract.

A. Duodenal adenoma B. Same adenoma as A. but in narrow band imaging (NBI) C. Adenoma at the major papilla in duodenum D. Duodenal adenoma with submucosal lifting before endoscopic mucosal resection (EMR) E. Endoscopic papillectomy with pancreatic stent F. Colonic polyposis.
