# Supplementary material for: Re-evaluating the genotypes of patients with adenomatous polyposis of unknown etiology: a nationwide study
Source: Eur J Hum Genet. 2024 Mar 12;32(5):588–92. doi: 10.1038/s41431-024-01585-z (PMC11061120; doi:10.1038/s41431-024-01585-z)
Supplement: Supplementary file 2 — Supplementary Material 2 [file 41431_2024_1585_MOESM2_ESM.docx]

| Genes involved in hereditary polyposis predisposition syndromes | | | |
| --- | --- | --- | --- |
| **Genetic syndrome and inheritance** | **Gene(s) involved** | **Histopathology of polyps** | **Cancer risk and non-malignant associated manifestations** |
| **Dominant inheritance** | | | |
| Familial adenomatous polyposis (FAP) | *APC* | Adenomas | Colorectal polyposis/CRC, duodenal cancer, hepatoblastoma, brain tumors, papillary thyroid cancer  Non-malignant features: Dental abnormalities, epidermoid cysts, desmoid tumours, osteomas and CHRPE*. |
| Oligodontia-colorectal syndrome | *AXIN2* | Mostly adenomas | Colorectal polyposis/CRC. Non-malignant feature: Tooth agenesis. |
| Juvenile polyposis | *BMPR1A*  *SMAD4* | Hamartomatous | Gastrointestinal hamartomatous polyps and CRC and other GI cancers. Non-malignant features (only *SMAD4*): Hereditary haemorrhagic telangiectasia and features of thoracic aortic aneurism/dissection. |
| Hereditary mixed polyposis syndrome | *GREM1* | Mixed-type | Colorectal polyposis/CRC |
| Polymerase proofreading-associated polyposis (PPAP) | *POLE, POLD1* | Adenomas | Colorectal polyposis/CRC, gynaecological cancers, brain tumours. Non-malignant features: Café-au-lait macules (rare). |
| Cowden syndrome / *PTEN* hamartoma tumor syndrome | *PTEN* | Hamartomatous | Hamartomatous gastrointestinal polyps and CRC, breast cancer, thyroid cancer, endometrial cancer, renal cancer.  Non-malignant features: Skin tumors, macrocephaly, vascular abnormalities, autism, intellectual disability. |
| Serrated polyposis syndrome | *RNF43*** | Serrated | Colorectal polyposis and CRC |
| Peutz-Jeghers syndrome | *STK11* | Hamartomatous | Polyposis (small intestine), GI cancer (CRC/gastric/pancreatic), breast cancer, ovarian cancer, cervical cancer.  Non-malignant features: Mucocutaneous pigmentation, benign ovarian/testicular tumors. |
| **Recessive inheritance** | | | |
| CMMR-D | *MLH1, MSH2, MSH6, PMS2* | Adenomas | Colorectal adenomas, GI cancer, brain tumors and haematological cancer. Non-malignant feature: Café-au-lait macules. |
| *MUTYH*-associated polyposis (MAP) | *MUTYH* | Adenomas | Colorectal adenomas/CRC, duodenal adenomas/cancer, other GI cancers, ovarian cancer, bladder cancer, skin cancer, late-onset breast cancer. |
| *NTHL1*-associated polyposis (NAP) / *NTHL1* tumour syndrome | *NTHL1* | Adenomas | Colorectal polyposis/CRC, breast cancer. Duodenal polyposis/cancer, endometrial cancer. |
| *MBD4*-associated neoplasia syndrome | *MBD4* | Adenomas | Colorectal polyposis/CRC, hematological cancer and other cancers. |
| *MSH3*-associated CRC | *MSH3* | Adenomas | Uncertain due to too few published cases. Suggested: Colorectal polyposis/CRC, gastric cancer. Benign tumors in thyroid, duodenum, brain, breast, uterus, ovaries. |
| *MLH3*-associated CRC | *MLH3* | Adenomas | Uncertain due to too few published cases. Suggested: Colorectal polyposis/CRC, breast cancer. |

*CHRPE = Congenital hypertrophy of the retinal pigment epithelium

** Pathogenic variants in *RNF43* are only detected in a subset of patients
